# Oncologist-Patient Concordance and Treatment Adherence in Chronic Myeloid Leukemia

**DOI:** 10.1001/jamanetworkopen.2025.8039

**Published:** 2025-04-30

**Authors:** J. Felipe Montano-Campos, Erin E. Hahn, Eric C. Haupt, Jerald Radich, Aasthaa Bansal

**Affiliations:** 1 Comparative Health Outcomes, Policy, and Economics Institute, School of Pharmacy, University of Washington, Seattle; 2 Department of Health Systems Sciences, Kaiser Permanente Bernard J. Tyson School of Medicine, Pasadena, California; 3 Department of Research and Evaluation, Kaiser Permanente Southern California, Pasadena; 4 Fred Hutchinson Cancer Center, Seattle, Washington; 5 Medical Oncology, University of Washington, Seattle

## Abstract

This cohort study investigates the association between oncologist-patient demographic concordance and adherence to tyrosine kinase inhibitor treatment in chronic myeloid leukemia.

## Introduction

Medical practice has increasingly emphasized relationship-oriented factors, such as cultural competence, trust, and effective communication, particularly in diverse patient populations.^1^ Beyond communication, shared identity between physicians and patients fosters deeper understanding, shaping clinical interactions and influencing health outcomes.^2^ This shared identity extends beyond individual relationships to broader structural factors, including societal status differences shaped by race and ethnicity, gender, and other cultural characteristics.

Patient-physician concordance, defined as demographic attributes shared between patients and physicians, has been identified as an important factor in reducing health care disparities and improving patient outcomes, particularly among minority populations.^3,4,5,6^ Given the importance of adherence to tyrosine kinase inhibitors (TKIs) in managing chronic myeloid leukemia (CML), we examined the association between oncologist-patient race and ethnicity and gender concordance and treatment adherence.

## Methods

We conducted a retrospective cohort study (2007-2019) using electronic health records from Kaiser Permanente Southern California (KPSC). The University of Washington Institutional Review Board (IRB) approved this study, with reliance from the KPSC IRB; the University of Washington Human Subjects Division waived informed consent because the study involved minimal risk and obtaining consent from all patients was not practical. We adhered to the STROBE reporting guideline. We included adults diagnosed with CML who were continuously enrolled in KPSC at diagnosis, excluding patients diagnosed more than 6 months before joining KPSC, those with an initial BCR-ABL1 measurement recorded more than a year after diagnosis, and those not prescribed TKIs. Additionally, we excluded patients who did not achieve a molecular response within 6 months of treatment initiation given that early nonresponse is a major driver of nonadherence.

We analyzed 3 primary exposures: oncologist-patient race and ethnicity concordance (shared racial and ethnic identity), gender concordance (shared gender identity), and combined race and ethnicity and gender concordance (shared racial and ethnic and gender identity). Extensive analysis was conducted to validate concordance metrics, including methodology for identifying the oncologist corresponding to each patient (eMethods in Supplement 1).

The main outcome was treatment adherence, measured using the Continuous Multiple-Interval Measure of Medication Availability 9 as a proportion based on pharmacy claims, tracking monthly adherence over 5 years. We used a linear mixed-effects model to assess associations between oncologist-patient concordance and adherence, adjusting for age, comorbidities, type of insurance, census-tract household income, physician experience, number of oncology visits, and number of physicians consulted in the first month. We incorporated random effects for patients and physicians to account for individual variability and clustering within oncologists, along with a random slope for the exposure to capture heterogeneity in its association with adherence.

## Results

The cohort included 443 patients (mean [SD] age, 53.6 [16.0] years; 39.1% female; 9.3% Asian, 10.2% Black, 36.6% Hispanic, and 42.4% non-Hispanic White). Among 117 oncologists (mean [SD] age, 50.7 [9.5] years; 47.8% female; 68.4% Asian, 2.6% Black, 3.4% Hispanic, and 22.2% non-Hispanic White), there was a mean (SD) of 14.9 (8.7) years of experience.

Among all patients, 17.2% had an oncologist of the same race and ethnicity, 51.4% had an oncologist of the same gender, and 11.4% had an oncologist matching both race and ethnicity and gender. For race and ethnicity concordance, adherence was higher by 5.0 percentage points (95% CI, −0.4 to 10.4% percentage points; *P* = .07), but this was not significant. Gender concordance was associated with 4.7–percentage point higher adherence (95% CI, 0.4 to 9.0 percentage points; *P* = .03), while combined race and ethnicity and gender concordance showed a 6.7–percentage point higher adherence (95% CI, 0.2 to 13.1 percentage points; *P* = .047). Adherence differences emerged early and persisted over time, highlighting sustained benefits associated with shared identity in clinical relationships (Table, Figure).

**Table.** Association of Patient-Oncologist Concordance With Treatment Adherence

Factor	Adherence difference, β (95% CI)^a^					
	Race and ethnicity^b^	*P* value^c^	Gender^b^	*P* value^c^	Race and ethnicity and gender^b^	*P* value^c^
Intercept	0.699 (0.555 to 0.842)	<.001	0.689 (0.546 to 0.833)	<.001	0.706 (0.562 to 0.850)	<.001
Concordance						
Race and ethnicity	0.050 (−0.004 to 0.104)	.07	NA	NA	NA	NA
Gender	NA	NA	0.047 (0.004 to 0.090)	.03	NA	NA
Race and ethnicity and gender	NA	NA	NA	NA	0.067 (0.002 to 0.131)	.047
Charlson Comorbidity Index score, per 1-unit increase	−0.008 (−0.020 to 0.003)	.16	−0.009 (−0.021 to 0.003)	.14	−0.008 (−0.020 to 0.003)	.16
Time, per 1-mo increase	0.001 (0.001 to 0.002)	<.001	0.002 (0.001 to 0.002)	<.001	0.002 (0.001 to 0.002)	<.001
Age, per 1-y increase	0.002 (0.000 to 0.004)	.03	0.002 (0.000 to 0.004)	.03	0.002 (0.000 to 0.004)	.04
Type of insurance						
Medicaid	0 [Reference]	NA	0 [Reference]	NA	0 [Reference]	NA
Medicare	−0.048 (−0.119 to 0.023)	.19	−0.053 (−0.124 to 0.018)	.15	−0.048 (−0.119 to 0.023)	.19
Commercial and private	−0.066 (−0.141 to 0.009)	.09	−0.071 (−0.146 to 0.004)	.07	−0.069 (−0.144 to 0.006)	.07
Physician experience, per 1-y increase	−0.001 (−0.004 to 0.001)	.47	−0.001 (−0.004 to 0.001)	.45	−0.001 (−0.004 to 0.001)	.46
Household annual income at baseline, per $10 000 increase	0.005 (−0.003 to 0.013)	.22	0.005 (−0.003 to 0.012)	.23	0.005 (−0.003 to 0.012)	.23
No. of visits in first mo	−0.099 (−0.562 to 0.363)	.68	−0.107 (−0.568 to 0.353)	.65	−0.094 (−0.557 to 0.368)	.69
No. of physicians consulted in first mo	−0.010 (−0.050 to 0.030)	.61	−0.010 (−0.050 to 0.030)	.62	−0.011 (−0.050 to 0.030)	.60

Abbreviation: NA, not applicable.

^a^ Associations are shown over a 5-year follow-up in a linear mixed-effects model incorporating patient and physician random effects and a random slope for concordance exposures.

^b^ Patient race and ethnicity and gender were obtained from electronic health records, where patients typically self-reported this information during their initial visit or enrollment. In some cases, health care professionals may have completed these fields during clinic visits. Oncologist race and ethnicity and gender data were extracted from the clinician database, which is recorded at the start of the oncologist’s employment at Kaiser Permanente Southern California. Race and ethnicity options were Asian, Black, Hispanic, and Non-Hispanic White. The information on race and ethnicity was included in the original dataset (eMethods in Supplement 1).

^c^ Statistical analyses were conducted June 2023 to July 2024 using R statistical software version 4.3.1 (R Project for Statistical Computing). A 2-sided *P* < .05 was considered statistically significant.

**Figure.**
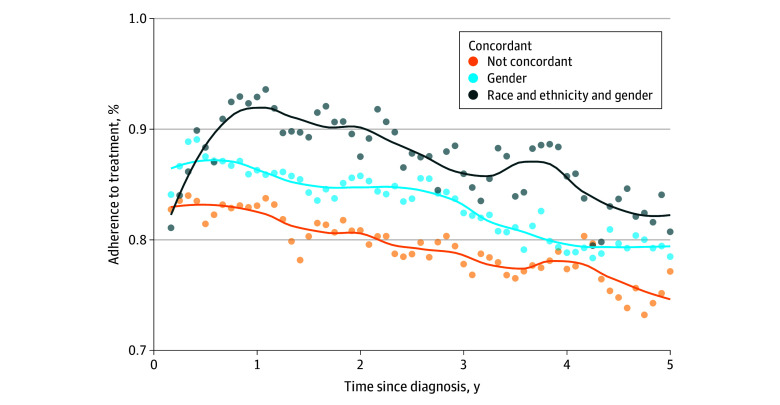
Treatment Adherence Trends by Race and Ethnicity and Gender Concordance

## Discussion

This cohort study’s findings highlight the role of oncologist-patient concordance in sustained treatment adherence over 5 years. By leveraging longitudinal clinical data, this study highlights how shared identity may be associated with long-term treatment behaviors. A limitation was that demographic and treatment characteristics, drawn from an integrated health care system, may not reflect all patients with CML, particularly those in other regions or health care settings. Increasing diversity in oncology through targeted recruitment, mentorship, and retention strategies may be associated with reduced adherence disparities, particularly for minority populations. Promoting concordant patient-physician relationships could further enhance adherence and engagement in care.

## Notes

Supplement 1.
eMethods.

Supplement 2.
Data Sharing Statement
