# Supplementary material for: Oncologist-Patient Concordance and Treatment Adherence in Chronic Myeloid Leukemia
Source: JAMA Netw Open. 2025 Apr 30;8(4):e258039. doi: 10.1001/jamanetworkopen.2025.8039 (PMC12044493; doi:10.1001/jamanetworkopen.2025.8039)
Supplement: Supplement 1. — eMethods. [file jamanetwopen-e258039-s001.pdf]

Supplemental Online Content

Montano-Campos JF, Hahn EE, Haupt EC, Radich J, Bansal A. Oncologist-patient concordance and treatment adherence in chronic myeloid leukemia. *JAMA Netw Open*. 2025;8(4):e258039. doi:10.1001/jamanetworkopen.2025.8039

eMethods.

This supplemental material has been provided by the authors to give readers additional information about their work.

© 2025 Montano-Campos JF et al. *JAMA Network Open*.

eMethods.

A. Data Sources for Patient and Provider Demographic Variables

Patient race/ethnicity and gender were obtained from electronic medical records, where patients typically self-report this information during their initial visit or enrollment. In some cases, healthcare professionals may complete these fields during clinic visits. Oncologist race/ethnicity and gender data were extracted from the provider database, which is recorded at the start of their employment at Kaiser Permanente Southern California (KPSC).

B. Checking for Bias in Exposure Definition and Consistency of Patient-Physician Relationship

While our exposure definition allows for time-varying concordance—since patients could see multiple oncologists during follow-up—most maintained consistent relationships with the same provider. To define the corresponding oncologist, we identified the physician with whom the patient had the highest number of visits from diagnosis through follow-up. Notably, 94% of patients consistently saw the same oncologist, meaning their primary oncologist at diagnosis remained their most frequently visited provider.

C. Examination of Concordance Metric Validity

© 2025 Montano-Campos JF et al. *JAMA Network Open*.

We considered potential endogeneity and confounding factors that could influence our observed associations. Prior research suggests that patient-physician race/ethnicity and gender concordance is often non-random. Patients may preferentially seek concordant physicians based on trust or comfort, while physician and patient characteristics could also contribute to selection bias [6]. These factors complicate efforts to isolate the true effect of concordance on adherence. To address this, we examined whether certain patient or physician characteristics were associated with concordance, assessing whether our concordance metric captured underlying patterns that may systematically correlate with specific attributes. We fitted three separate linear regression models, each using one of the three concordance metrics as the outcome variable. Predictors included patient age, Charlson comorbidity index, insurance type and coverage, census-tract household income, visit frequency, physician experience, and the number of different physicians a patient consulted during follow-up. The latter was particularly important, as patients may seek multiple physicians to find one that aligns with their preferences. Results showed that only census-tract household income was positively associated with a higher likelihood of any type of concordance (adjusted for in our primary analysis). Other predictors did not significantly influence the probability of concordance.

© 2025 Montano-Campos JF et al. *JAMA Network Open*.
