# Supplementary material for: Oncologist-Patient Concordance and Treatment Adherence in Chronic Myeloid Leukemia
Source: JAMA Netw Open. 2025 Apr 30;8(4):e258039. doi: 10.1001/jamanetworkopen.2025.8039 (PMC12044493; doi:10.1001/jamanetworkopen.2025.8039)
Supplement: Supplement 2. — Data Sharing Statement [file jamanetwopen-e258039-s002.pdf]

Data Sharing Statement

Montano-Campos. Oncologist-Patient Concordance and Treatment Adherence in Chronic Myeloid Leukemia. *JAMA Netw Open*. Published online April 30, 2025. doi:10.1001/jamanetworkopen.2025.8039

Data
Data available: No

Additional Information
Explanation for why data not available: The data are restricted by a Data Use Agreement with Kaiser Permanente
